# Prevalence and Indicators of Vitamin B12 Insufficiency among Young Women of Childbearing Age

**DOI:** 10.3390/ijerph18010001

**Published:** 2020-12-22

**Authors:** Sara Al-Musharaf, Philip G. McTernan, Syed Danish Hussain, Khalid Abdullah Aleisa, Abdullah M. Alnaami, Kaiser Wani, Ponnusamy Saravanan, Nasser Al-Daghri

**Affiliations:** 1 Department of Community Health Sciences, College of Applied Medical Sciences, King Saud University, Riyadh 11451, Saudi Arabia; 2 Chair for Biomarkers of Chronic Diseases, Riyadh Biochemistry Department, College of Science, King Saud University, Riyadh 11451, Saudi Arabia; danishhussain121@gmail.com (S.D.H.); aalnaami@yahoo.com (A.M.A.); kwani@ksu.edu.sa (K.W.); ndaghri@ksu.edu.sa (N.A.-D.); 3 Department of Biosciences, School of Science and Technology, Nottingham Trent University, Nottingham NG1 8NS, UK; 4 College of Medicine, King Saud University, Riyadh 11451, Saudi Arabia; Aleisa.Khaled@gmail.com; 5 Population, Evidence & Technologies, Division of Health Sciences, Warwick Medical School, University of Warwick, Coventry CV2 2DX, UK; p.saravanan@warwick.ac.uk; 6 Academic Department of Diabetes, Endocrinology & Metabolism, George Eliot Hospital, Nuneaton CV10 7DJ, UK

**Keywords:** women, sedentary, physical activity, vitamin B12 insufficiency, vitamin B12

## Abstract

Vitamin B12 insufficiency is a global health issue among women of childbearing age, yet few studies have investigated its prevalence and risk factors among healthy Middle Eastern populations. This cross-sectional study included 346 Saudi women aged 19–30 years and enrolled at King Saud University, Riyadh, Saudi Arabia. A series of questionnaires were administered to record the study participants’ sociodemographic status, medical history, dietary intake, and physical activity. Participants’ anthropometric data were also recorded and their fasting blood samples were analyzed. The rate of vitamin B12 insufficiency (≤220 pmol/L) was approximately 6% among the study participants. After adjusting for confounding factors, it was observed that the risk factors for vitamin B12 insufficiency included daily sitting time ≥ 7 h, low income (<10,000 Saudi riyal) and increasing age. The recommended dietary allowance of vitamin B12 (>2.4 mcg/day) has been shown to confer reasonable protection against vitamin B12 insufficiency. These study findings highlight that a combination of increased physical activity and dietary vitamin B12 intake above the current recommended dietary allowance may help improve the serum vitamin B12 levels of young women of childbearing age, especially those with a low socioeconomic status. Timely detection and protection against vitamin B12 insufficiency in this subpopulation are important to prevent maternal and fetal health risks.

## 1. Introduction

Vitamin B12 is an essential micronutrient required for the biological synthesis of macronutrients, red blood cells, and DNA [1]. It must be obtained from dietary sources such as foods from animal origin, seaweed and fermented vegetables, as the human body cannot synthesize it [2,3]. The bioavailability of B12 in the body depends on the type and amount of its intake from food [1], and as the most prominent intake is from animal products, vegans and vegetarians are at risk of low B12 intake and subsequent dietary deficiency [2]. Deficiency may also result from impaired vitamin B12 absorption due to a lack of hydrochloric acid and/or intrinsic factors [3]. Vitamin B12 deficiency is associated with serious health problems, ranging from mild fatigue and anemia to severe neurological dysfunction [3,4], osteoporosis [5], and metabolic diseases [6,7], as well as an increase in the biochemical markers of disease risk [8]. Further to this, epidemiological studies indicate that vitamin B12 deficiency is associated with obesity, gastrointestinal disease, bariatric surgery, and renal insufficiency [9,10,11]. Studies have also revealed an association between low vitamin B12 status and certain ethnic groups [12], low income [13], and lifestyle factors, such as higher consumption of alcohol [9], caffeine, or tobacco [14], and a sedentary lifestyle [9]. The regular use of certain medications, such as metformin [15] and proton pump inhibitors [16], may also cause vitamin B12 deficiency. As vitamin B12 deficiency is generally considered a problem among elderly people [17], its occurrence in young adults has received less attention.

The prevalence rates of vitamin B12 deficiency range from 2.5% to 60%, differing by age group, gender, and ethnicity [8,18,19]. While most studies have used <148 pmol/L as the cut-off to define vitamin B12 deficiency [8,19,20], some studies proposed a higher concentration (≤220 pmol/L) based on homocysteine and methyl malonic acid levels, which are tissue markers of vitamin B12 deficiency [8,12,21]. According to the most recent National Health and Nutrition Examination Survey in the United States, vitamin B12 deficiency among people over the age of 19 in the general population ranges from 3% to 26% depending on the cut-off used, with women more likely to be deficient than men [19]. A Canadian study on healthy, non-pregnant women aged 19–35 (n = 206) and of South Asian or European ethnicity reported a 34% prevalence of vitamin B12 insufficiency at the <220 pmol/L threshold [12]. We used both cut-offs in this study to facilitate comparisons with other studies.

The World Health Organization (WHO) identifies pregnant and lactating women as among the groups at greatest risk of vitamin B12 deficiency [22]. This is problematic, as during pregnancy vitamin B12 deficiency is associated with gestational diabetes mellitus [23], infant health issues stemming from intrauterine growth restriction [24], impaired cardiometabolic health of the fetus [25], and an increased risk of the fetus suffering developing congenital anomalies [26]. Among the rapid social and cultural changes throughout the Middle East, including Saudi Arabia, is that young women in particular are increasingly concerned with beauty, body image, and vegetarian diets to support good health, putting them at increased risk of having vitamin B12 insufficiency [27]. Research has revealed that at the time of the birth of their first child, approximately 80% of Saudi women have an average age of 20–25 years [28] and around 50% have a college education degree [28,29].

Evaluating the risk of having vitamin B12 insufficiency (≤220 pmol/L) in young Saudi women attending university is important, as the average age of university-going female students in Saudi Arabia coincides with the average age of the childbearing female population. Furthermore, a recent systematic review of studies on pregnant women indicated vitamin B12 insufficiency rates of 20–30% during all three trimesters and higher rates in some ethnic groups [30]. Therefore, it is important to estimate the extent of vitamin B12 insufficiency in this population subgroup and to prevent vitamin B12 deficiency and its subsequent adverse health effects in both pregnant and non-pregnant women.

There has been limited research on the prevalence of vitamin B12 insufficiency (≤220 pmol/L) in Middle Eastern adults, especially in apparently healthy young women of childbearing age. The few existing studies cover both sexes, a wide age range, and unhealthy populations [20,31]. To the best of our knowledge, no studies have been conducted on the prevalence of vitamin B12 insufficiency in healthy young women in the Middle East. It is necessary to research vitamin B12 insufficiency levels in this population in the Middle East, as about half of the pregnancies in this population are unplanned, even in the developed countries of the region [32]. Furthermore, approximately 70% of the pregnant women in Saudi Arabia are between the ages of 20 and 30 [33]. In this study, we investigated the prevalence of vitamin B12 insufficiency in apparently healthy women between 19 and 30 years of age. We also explored the likely predictors of vitamin B12 status, such as sociodemographic characteristics, dietary intake, and physical activity.

## 2. Materials and Methods

### 2.1. Study Population

This study was an observational, cross-sectional study of Saudi female college students aged 19–30 years. The central university has students coming from all parts of the country and is therefore representative of the young women from the whole country. This study formed part of a parent study for which ethical approval was obtained from the Institutional Review Board (IRB) of King Khalid University Hospital, Riyadh (IRB No. E-19-3625).

### 2.2. Inclusion and Exclusion Criteria

The study participants satisfied the following criteria: Female, generally in good health, Saudi national, and enrolled at King Saud University (KSU). The prospective study participants who were not female or Saudi nationals, or who were pregnant, lactating, or had previously been diagnosed with a gastrointestinal disorder, significant proteinuria or amyloidosis, anemia, malabsorption, a metabolic disorder, or a comorbid chronic disease (e.g., thyroid disorder, diabetes mellitus, malignancy, chronic obstructive pulmonary disease) were excluded from the study. The prospective study participants who were taking vitamin B12 supplements or medication with known effects on the vitamin B12 serum concentration were also excluded from the study. Only women who consented to participate in the study were included in the study.

### 2.3. Sample Size

KSU is one of the largest public universities in Saudi Arabia, with more than 50,000 students. The university student population in Saudi Arabia can be considered representative of the country’s young population. The average age of the study participants was 20.7 ± 1.5 years, and most came from families with an average monthly income of more than SAR 10,000. Thus, they were also representative of the young women in Saudi Arabia in terms of age and socioeconomic status [34]. Assuming an expected 22.7% vitamin B12 insufficiency (≤220 pmol/L) prevalence among Saudi women [35], a 5% significance level and a 5% precision level, and considering that the number of young Saudi women (aged 20–29) is 454,830 [36], the required sample size was 269, calculated using Statcalc (EPI InfoTM). To account for the potential non-responses, we randomly selected 355 women from different departments at KSU as prospective study participants. Of these, 346 provided us with all of the information about them that we needed and were included in the data analysis.

### 2.4. Assessment of Vitamin B12 Status

Blood samples were collected from all of the study participants and were sent to the Prince Mutaib bin Abdullah Chair at KSU for analysis. All of the samples were aliquoted and frozen at −80 °C for further chemical analysis at KSU’s Chair for Biomarkers of Chronic Diseases laboratories.

The serum vitamin B12 concentration was determined through electrochemiluminescent immunoassays using a Roche Cobas e411 immunoassay analyzer (Roche Diagnostics, Germany). In accordance with the previously published studies, we defined vitamin B12 deficiency as a serum vitamin B12 concentration lower than 148 pmol/L (200 pg/mL), and vitamin B12 insufficiency (borderline deficiency) as a serum vitamin B12 concentration of 148–221 pmol/L (200–300 pg/mL) [8,13,21,37]. The intra- and inter-assay coefficient-of-variation values were 2.9% and 4.1%, respectively.

### 2.5. Anthropometric Assessment

Anthropometric data were collected using standard procedures. Weight and height were recorded to the nearest 0.2 kg and 0.5 cm, respectively, using an appropriate international standard scale (Digital Pearson Scale, ADAM Equipment Inc., Oxford, CT, USA). The weight (kg) was divided by the square of the height (m^2^) to calculate the body mass index (BMI; kg/m^2^). The BMI cut-offs used by the WHO were categorized into four groups: underweight (<18.5 kg/m^2^), normal weight (18.5–24.9 kg/m^2^), overweight (25.0–29.9 kg/m^2^), and obese (≥30 kg/m^2^) [38]. The circumference of the waist and hip was measured according to the procedure outlined by the WHO [38]. The waist-to-hip ratio (WHR) was obtained by dividing the mean waist circumference by the mean hip circumference. A Body 770 body composition analyzer (Cerritos, CA, USA) was used to assess the body fat percentage.

### 2.6. Socioeconomic Status and Lifestyle Assessment

Each study participant was interviewed to complete a general questionnaire [39] concerning their sociodemographic information (i.e., college attended, major specialty, major level, family income, type of residence and living location, family medical history, and own medical history) and medication treatment history. Participants’ income was based on the income of their parents.

### 2.7. Dietary Data

The Saudi Food and Drug Administration’s food frequency questionnaire (SFDA FFQ) was validated and used to assess the study participants’ dietary intake in the preceding year [40]. The Arabic-language questionnaire has 133 questions related to food. The frequency-of-consumption choices are stated as follows: never or less than once per month, one to three times per month, once per week, two to four times per week, five to six times per week, once per day, two to three times per day, four to five times per day, and six or more times per day. The questionnaire also asks about the food items consumed by the respondents other than those listed, and includes questions concerning how the respondents cook fat, their visible-fat consumption, their consumption of salt and vitamins, and their protein supplementation [40]. The SFDA FFQ responses were analyzed using a Microsoft Excel spreadsheet provided by Dr. Majed Alkhalaf (Saudi Food & Drug Authority, Saudi Arabia). The item values were based on a 1996 Saudi food composition table, the 7th edition of McCance and Widdowson’s Composition of Foods Integrated Dataset and the 12th edition of the Concise New Zealand Food Composition Tables [40,41,42]. A second validated questionnaire was adopted specifically to measure vitamin B12 intake from food and beverages [42]. The recommended dietary allowance (RDA) of vitamin B12 for adults suggested by many guidelines is 2.4 mcg/day [3], as this has been shown to confer reasonable protection against vitamin B12 insufficiency, although a higher amount might be needed [43].

### 2.8. Physical Activity Estimates

The Global Physical Activity Questionnaire (GPAQ) version 2.0 measures several components of physical activity and includes intensity and frequency of physical activity. It also assesses three domains of physical activity: Occupational physical activity, transport-related physical activity, and physical activity during discretionary or leisure time. The validated Arabic-language version of GPAQ that was previously used on a college-age Saudi sample population was used in this study [44]. Further analysis was conducted to define sedentary students as those sitting for more than 390 min/d (≥7 h/d) [45,46]. Vigorous physical activity was presented both continuously and categorically (>60 min) to facilitate analysis.

### 2.9. Statistical Analysis

The data were analyzed using SPSS version 23.0. The normality of each quantitative variable was tested before the analysis. Descriptive statistics (mean, standard deviation, median, quartile, frequency, and percentage) were used to quantify the quantitative and categorical variables. Associations between continuous variables were identified using Pearson’s correlation coefficient, and associations between categorical variables were identified using Pearson’s chi-squared test of independence. Student’s *t*-test for independent samples was also used. Appropriate non-parametric tests were used if variables were non-normally distributed. Odds ratios (ORs) and 95% confidence intervals for these were obtained using multivariate binary logistic regression analysis, taking vitamin B12 insufficiency (cut-off ≤ 220 pmol/L) as a dependent variable to identify its potential risk factors. Results were considered statistically significant at *p* < 0.05.

## 3. Results

The study group consisted of 346 Saudi women with a mean age of 20.7 ± 1.5 years. Using the definition of family income mentioned in the Materials and Methods section, 20.8% of the study participants had a monthly income of less than 10,000 Saudi riyal. In addition, approximately one-third (39%) of the study participants resided in northern Riyadh, and most (97.2%) were single at the time of the study. More socio-demographic data are shown in supplementary Table S1.

The average BMI, fat percentage, and WHR of the study participants were 23.6 ± 5.2 kg/m^2^, 36.9% ± 8.2%, and 0.7 ± 0.1, respectively. The median, 25th percentile and 75th percentile vitamin B12 serum concentrations were 399 (306–535) pmol/L. The median total time of physical activity per week was 504 (160–1240) min. The median sitting time per day was 420 (240–600) min (7 h). As shown in Table 1, the median vitamin B12 intake was 6.9 (4.4–10.8) mcg/d.

### 3.1. Prevalence of Vitamin B12 Insufficiency (≤220 pmol/L)

The prevalence of vitamin B12 serum deficiency, insufficiency, and sufficiency was determined using the following cut-off points: <148, 148–220, and ≥221 pmol/L. The results indicated 0.6% (2/346) serum vitamin B12 deficiency, 5.5% (19/346) serum vitamin B12 insufficiency, and 93.9% (325/346) serum vitamin B12 sufficiency.

When the ≤220 pmol/L serum vitamin B12 level was used as a cut-off for analysis, the level of vitamin B12 insufficiency was approximately 6% (21/346). We further analyzed the data to see if there were any differences in the serum vitamin B12 status of the study participants from different departments, and we found no statistically significant differences (*p* < 0.05; not shown in Table 1).

### 3.2. Baseline Characteristics by Vitamin B12 Status

Age (in years) did not significantly differ between the study groups based on their vitamin B12 status (*p* = 0.312). The study participants’ socioeconomic data were presented as valid percentages of all the demographic variables (Table 1). No significant sociodemographic differences were observed among the vitamin-B12-insufficient study participants in terms of income level, living area, place of residence, or civil status compared to the vitamin-B12-sufficient group.

**Figure 1:**
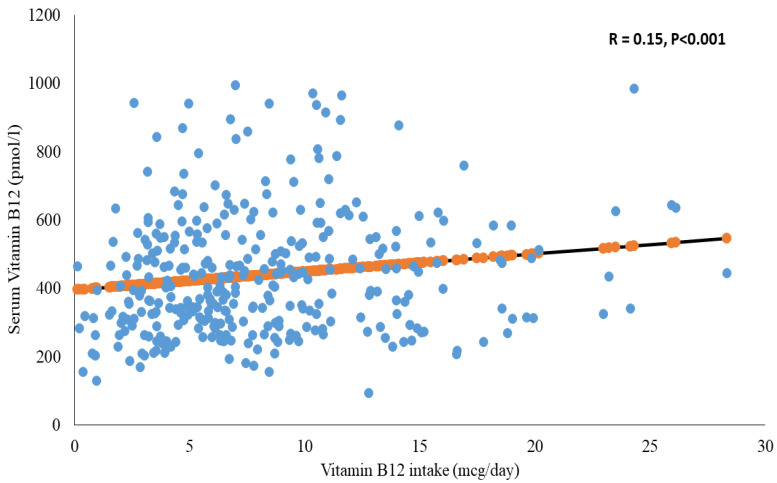
Correlation between vitamin B12 intake and vitamin B12 levels.

The median vitamin B12 intake of the vitamin-B12-sufficient group was significantly higher than the vitamin-B12-insufficient group (7.0 vs. 3.9 mcg/day; *p* = 0.01). Furthermore, the vitamin B12 intake was found to be significantly positively correlated with the vitamin B12 serum concentration using Spearman’s correlation test (*r* = 0.15, *p* < 0.05; Figure 1). Most study participants (92.4%) had vitamin B12 intake levels above the RDA of 2.4 mcg/d, but only 17 of the 21 vitamin-B12-insufficient study participants had estimated vitamin B12 intake levels above the RDA (Table 1). There were no differences in total caloric intake and in macronutrients/micronutrients status between the vitamin-B12-insufficient and vitamin-B12-sufficient groups. Interestingly, the analysis of the vitamin-B12-insufficient group revealed that there were more people in the group who were taking protein and multivitamin supplements than in the vitamin-B12-sufficient group (4.8% vs. 0.3%). However, this difference was not statistically significant (Table 1).

The anthropometric characteristics and vitamin B12 concentrations of the vitamin-B12-sufficient and vitamin-B12-insufficient groups are shown in Table 1. No differences were noted between the two groups with respect to the BMI, WHR, and fat percentage. The median vitamin B12 concentrations in the vitamin-B12-sufficient and vitamin-B12-insufficient groups were 425.6 and 203.4 pmol/L, respectively.

With regard to physical activity, there was a higher prevalence of vitamin B12 insufficiency (66.7%) among the study participants who remained seated for at least 7 h/d than among those who remained seated for <7 h/d (49.2%), but this difference was not significant (*p* = 0.121). Interestingly, vigorous physical activity was significantly positively associated with vitamin B12 intake (*r* = 0.13, *p* = 0.01). No significant differences in the GPAQ total scores were found between the vitamin-B12-insufficient and vitamin-B12-sufficient groups (*p* = 0.72), as shown in Table 1.

### 3.3. Indicators of Vitamin B12 Insufficiency (≤220 pmol/L)

The risk factors for vitamin B12 insufficiency among the study participants are presented in Table 2. The study results showed that a vitamin B12 intake greater than the RDA of 2.4 mcg/d reduces the odds of vitamin B12 insufficiency by 18% (*p* = 0.039), and that higher age, lower family income, and sedentary behavior are associated with a higher risk of vitamin B12 insufficiency. Furthermore, when categorizing the data into vitamin B12 serum quartiles, the lowest quartile had a cutoff of 305.8 pmol/l and included a majority of subjects with sufficient levels of vitamin B12, thus reducing the likelihood of finding significant effects among groups (Supplementary Table S2). 

## 4. Discussion

To the best of our knowledge, this is the first study to assess the prevalence of vitamin B12 insufficiency (≤220 pmol/L) and its risk factors among apparently healthy young female university students in the Middle East. Research has revealed that 80% of Saudi women have an average age of 20–25 years at the time of the birth of their first child [28]. Past research data also revealed that about half of Saudi women have a college education degree at the time of the birth of their first child [28,29]. The current study participants were females from 19 to 30 years old. They were thus representative of the young women of childbearing age in Saudi Arabia, as the average age of Saudi women at the time of their first marriage is 19.71 ± 3.77 years and their mean age at the birth of their first child is 21.1 ± 3.6 years [47]. The vitamin B12 status of this young female population subset is important because these women are likely to become mothers in the future and their nutritional status during pregnancy can have implications on the nutritional status and health of their children at birth.

Our findings have shown that the level of vitamin B12 insufficiency in the study cohort was approximately 6% (serum vitamin B12 ≤ 220 pmol/L), with 5.5% showing a serum vitamin B12 concentration of 148–220 pmol/L. There has been a call to identify the functional consequences of borderline vitamin B12 insufficiency [13], which is especially relevant for women of childbearing age to ensure the optimal health of infants and children.

The vitamin B12 insufficiency (≤220 pmol/L) of the healthy young women in our study ranged from 0.6% to 6%, depending on the cut-off point adopted. Few studies have assessed the vitamin B12 status of healthy Middle Eastern populations. A Jordanian survey of varying age and gender showed that 30.1% of women aged ≥ 19 years were deficient in vitamin B12 (<147 pmol/L) [20], while a Turkish study of non-pregnant women aged 18–45 years noted that the prevalence of vitamin B12 insufficiency among them was lower, at 22% (≤220 pmol/L) [48]. The reason for the difference between our findings and those studies may be because they were conducted in regions with populations that are at a high risk of developing thalassemia and where there is a significant minority (24.9%) of women with anemia [48]. The differences between the two aforementioned studies could be due to the nature of the studies’ sample populations. In our sample population, the median vitamin B12 intake of the participants was 6.9 mcg/d, and around 92% of the study participants consumed more than the RDA of >2.4 mcg/d. Previous studies have indicated that a dietary vitamin B12 intake of 5–10 mcg/d ensures maximal plasma vitamin B12 concentrations in healthy adults with adequate vitamin B12 absorption [43,49,50]. Despite their apparently adequate intake of vitamin B12, 17 of the 21 women in this study had vitamin B12 insufficiency (≤220 pmol/L). Therefore, it may be advisable to suggest RDA for vitamin B12 to be at least 5 mcg/d. The underlying factors for this higher prevalence, despite an apparently adequate intake, could be due to changes in the dietary habits of the Saudi women studied, which include unhealthy food consumption patterns. These dietary changes include both extremely unhealthy consumption of junk foods [51] and a trend toward strict diets, including vegetarianism, resulting in vitamin B12 insufficiency [27]. Furthermore, addressing the issue of vitamin B12 insufficiency (≤220 pmol/L) is expected to have a positive impact on the nutritional status of not only the subset of women who are likely to become mothers in the future, but also in young women, indicating that an appropriate dietary and/or supplementary method for addressing vitamin B12 insufficiency (≤220 pmol/L) should be adopted by this group.

After adjusting for all of the confounding factors, it was observed that the key risk factors for vitamin B12 insufficiency (≤220 pmol/L) were daily sedentary behavior, low income, and age. Unlike in other studies [52], vitamin B12 insufficiency was not observed to be associated with obesity in our study. Recent studies explored the relationship between vitamin B12 status and physical activity but they yielded conflicting results. Our study showed that the average Saudi woman sits for around 7 h/d; thus, the average Saudi woman can be considered sedentary [46]. Women sitting for longer periods of time are 4.6 times more likely to develop vitamin B12 insufficiency (≤220 pmol/L) compared with women with higher levels of activity.

While few studies have investigated the association between vitamin B12 levels and a sedentary lifestyle, a prior study has reported that individuals with active lifestyles, specifically those engaging in longer durations of regular exercise, displayed significantly higher vitamin B12 concentrations than the non-exercise group [53]. However, other studies have not identified any relationship between vitamin B12 status and physical activity [54,55]. It must be noted that the conflicting results in these studies may be attributed to differences in their inclusion criteria, including wider age ranges (e.g., elderly people) and/or a very small population of highly active women.

There are several potential explanations for the association between vitamin B12 levels and physical activity. For one, a sedentary lifestyle increases the homocysteine level through different pathways, resulting in a lower serum vitamin B12 level [56]. Another potential explanation for the observed increase in vitamin B12 levels with increased activity is the association of increased activity with increased food intake, which may boost the body’s supply of B vitamins [57]. This was clear in the present study—vigorous physical activity was positively correlated with vitamin B12 intake, and the findings are concordant with those of a previous study, which showed that highly active women tend to have a higher vitamin B12 intake [54].

In this study, we observed that women who were from low-income families had lower vitamin B12 concentrations and a higher risk of vitamin B12 insufficiency, similar to findings of previous research [13,21,48]. This association could be attributed to the limited availability and affordability of animal-based foods amongst low- and middle-income people [58]. While Saudi Arabia is considered a rich country, 12.7–25% of its citizens are considered poor [59].

In this study, there was no noted association between low-income status and sedentary behavior. However, other studies have shown that these two factors are related [9,13], potentially due to low-income people less access to foods rich in vitamin B12.

Although our sample population was young (19–30 years old), increasing age even within this group increased the risk of an observed vitamin B12 insufficiency (≤220 pmol/L). In our study, vitamin B12 insufficiency was noted to increase by 38% with each one-year increase in age. The association of age with vitamin B12 concentration is well established, especially among older people [19,21]. A past longitudinal study on maternal and fetal vitamin B12 levels demonstrated that maternal vitamin B12 levels decrease by around 10–20% from preconception to early pregnancy [60], highlighting the importance of optimizing the vitamin B12 concentration, which is likely to experience a further drop if a woman in this age group becomes pregnant [22]. This highlights the need to pay greater attention to older and low-income women, and to advise these at-risk populations to undergo a vitamin B12 assessment if they are planning to become pregnant.

Our study had important limitations. First, it used a cross-sectional design, which precluded causal inference by ascertaining the directionality of relationships. Second, although plasma vitamin B12 measurement is a suitable means of assessing general vitamin B12 status in population surveys [61], plasma methylmalonic acid, homocysteine, or holotranscobalamin II can also be used for a more sensitive evaluation of vitamin B12 status. Finally, recall bias may occur during data collection through questionnaires (in the case of this study, the FFQ and GPAQ) which depend on subjective reporting as in all studies of this nature.

## 5. Conclusions

In summary, this study showed that 6% of apparently healthy young Saudi female university students, representative of the Saudi women of childbearing age, had vitamin B12 insufficiency (≤220 pmol/L), despite adequate vitamin B12 intake. The risk factors for vitamin B12 insufficiency were noted to be a sedentary lifestyle, age, and a lower socioeconomic status. These associations amongst age, socioeconomic class, and vitamin B12 insufficiency need to be investigated in future studies to conclusively demonstrate the relationships among these variables. This study highlighted the importance of measuring the vitamin B12 levels of women of childbearing age, especially if they are planning to become pregnant. This may help improve the vitamin B12 status of such women, which in turn can help decrease the adverse effects of vitamin B12 insufficiency in young women and their offspring.

## Figures and Tables

**Table 1:** Baseline characteristics in relation to vitamin B12 status.

	Overall	Insufficient (≤220 pmol/L)	Sufficient (>220 pmol/L)	*p*
**N**	346	21	325	
**Anthropometrics**				
Age (y)	20.7 ± 1.5	21.2 ± 2.2	20.7 ± 1.5	0.14
BMI (kg/m^2^)	23.6 ± 5.2	23.8 ± 5.4	23.6 ± 5.2	0.92
Waist-to-hip ratio	0.7 ± 0.1	0.7 ± 0.1	0.7 ± 0.4	0.96
Fat (%)	36.9 ± 8.2	37.4 ± 9.0	37.0 ± 8.0	0.80
**Socio-demographics**				
Income (<10K SAR/m)	74 (20.8)	7 (33.3)	63 (19.4)	0.16
Residence				
North Riyadh	136 (39.0)	6 (30.0)	127 (39.7)	0.23
West Riyadh	62 (17.8)	1 (5.0)	59 (18.4)	
East Riyadh	81 (23.2)	7 (35.0)	70 (21.9)	
South Riyadh	52 (14.9)	5 (25.0)	47 (14.7)	
Central Riyadh	18 (5.2)	1 (5.0)	17 (5.3)	
**Dietary**				
Fat (g/d)	125.1 (82.7–178.5)	107.4 (88.6–162.2)	125.5 (82.7–175.5)	0.61
Protein (g/d)	105.3 (78.0–141.8)	93.3 (79.7–143.4)	107.1 (79.3–140.0)	0.41
Carbohydrate (g/d)	367.6 (267.7–478.7)	356.7 (260.5–518.4)	368.5 (268.4–481.5)	0.91
Fiber (g/d)	29.8 (20.2–41.7)	35.0 (23.4–52.0)	29.5 (20.2–41.2)	0.24
Energy (kcal/d)	2918.6 (2133–3810)	2467.8 (2294.9–3820.3)	2941.0 (2132.5–3774.5)	0.83
Tea	103.2 (33.6–240.0)	103.2 (33.6–240.0)	103.2 (33.6–240.0)	0.60
Caffeine	105.0 (38.7–210.0)	135.0 (64.4–285.0)	103.2 (38.7–210.0)	0.21
VB12 (mcg/d)	6.9 (4.4–10.8)	3.9 (2.8–7.9)	7.0 (4.7–10.9)	**0.01**
VB12 intake ≥ 2.4 mcg/d	328 (92.4)	17 (81.0)	303 (93.2)	**0.04**
Protein supplement	12 (3.5)	1 (4.8)	11 (3.4)	0.53
Multivitamin supplement	2 (0.6)	1 (4.8)	1 (0.3)	0.12
**Physical Activity**				
Vigorous (min/w) ^#^	0.0 (0.0–0.0)	0.0 (0.0–0.0)	0.0 (0.0–0.0)	0.75
MET vigorous (min/w)	0.0 (0.0–0.0)	0.0 (0.0–0.0)	0.0 (0.0–0.0)	0.75
Vigorous (≥60 min/w)	62 (18.0)	3 (14.3)	59 (18.2)	1.00
Moderate (min/w) ^#^	90.0 (30.0–215.0)	135.0 (60.0–175.0)	90.0 (30.0–212.0)	0.35
MET moderate (min/w)	360.0 (120.0–860.0)	540.0 (240.0–700.0)	360.0 (120.0–848.0)	0.35
Sitting (min/d) ^#^	420.0 (240.0–600.0)	480.0 (360.0–600.0)	360.0 (240.0–600.0)	0.33
Sitting (≥7 h/d)	174 (50.3)	14 (66.7)	160 (49.2)	0.12
GPAQ (MET-min/w) ^#^	504.0 (160.0–1240.0)	660.0 (428.0–960.0)	480.0 (160.0–1280.0)	0.72
**Biochemical Data**				
Glucose (mmol/L)	4.6 ± 1.0	4.7 ± 1.1	4.6 ± 1.0	0.87

Data are presented as means ± SDs for normal variables and medians (first quartile–third quartile) for non-normal variables. ^#^ indicates non-normal variables. P values were obtained using the independent sample t-test and the Mann–Whitney U test for normal and non-normal variables, respectively. N (%) is used for categorical variables. P values for categorical variables were obtained using Pearson’s chi-square and Fisher’s exact test. *p* < 0.05, indicated in bold, was considered significant. MET: Metabolic equivalent; GPAQ: Global Physical Activity Questionnaire.

**Table 2:** Indicators of vitamin B12 insufficiency (≤220 pmol/L) among women of childbearing age.

Parameter	OR (95%CI)	*p* Value
Age (y)	1.38 (1.04–1.83)	**0.02**
BMI (kg/m^2^)	1.01 (0.92–1.10)	0.85
Glucose level (>5.6 mmol/L)	0.86 (0.17–4.33)	0.85
Vitamin B12 intake (RDA > 2.4 mcg/d)	0.10 (0.02–0.63)	**0.01**
Using protein supplement	2.94 (0.27–32.29)	0.38
Protein intake (>46 g/d)	3.03 (0.20–46.63)	0.42
Coffee intake (>750 mL/d)	2.04 (0.17–24.68)	0.58
Income (<10,000 Saudi riyal)	4.13 (1.25–13.64)	**0.02**
Sitting time (≥7 h/d)	4.60 (1.31–16.21)	**0.02**
Vigorous physical activity (≥60 min/w)	0.52 (0.10–2.72)	0.44

ORs and 95% CIs for ORs were obtained using multivariate logistic regression analysis, taking vitamin B12 insufficiency (≤220 pmol/L) as a dependent variable against potential risk factors and as an independent risk. A *p* value < 0.05, indicated in bold, was considered significant.

## Data Availability

Data from this study are available from the corresponding author on reasonable request.

## Supplementary Materials

The following are available online at https://www.mdpi.com/1660-4601/18/1/1/s1, Table S1: Participants’ sociodemographic characteristics according to vitamin B12 status and Table S2: Predictors of vitamin B12 serum by quartiles among women of childbearing age.

## Abbreviations

BMI: Body mass index; GPAQ: Global Physical Activity Questionnaire; IRB: Institutional review board; KSU: King Saud University; ORs: Odds ratios; RDA: Recommended dietary allowance; SFDA FFQ: Saudi Food and Drug Administration’s food frequency questionnaire; WHR: Waist-to-hip ratio; WHO: World Health Organization.
