# Supplementary material for: Prevalence and Indicators of Vitamin B12 Insufficiency among Young Women of Childbearing Age
Source: Int J Environ Res Public Health. 2020 Dec 22;18(1):1. doi: 10.3390/ijerph18010001 (PMC7792587; doi:10.3390/ijerph18010001)
Supplement: Supplementary file 1 [file ijerph-18-00001-s001.pdf]

Table 1. Participants' sociodemographic characteristics according to vitamin B12 status.

	Overall	Insufficient (≤ 220 pmol/L)	Sufficient (> 220 pmol/L)	***P***
**N (%)**		21 (6.1)	325 (93.9)	
**Socio-demographic data**				
**Department**				
Medical	114 (32)	4 (19)	107 (33)	
Science	118 (33)	7 (33)	108 (33)	0.32
Humanities	123 (35)	10 (48)	110 (34)	
**College level**				
< 5 semesters completed	189 (53)	12 (57)	173 (53.2)	0.73
≥ 5 semesters completed	166 (47)	9 (43)	152 (46.8)	
**Housing**				
Apartment	26 (7.6)	0 (0.0)	26 (8.0)	
Villa	300 (88)	19 (95)	281 (87)	0.57
Women' housing	15 (4.4)	1 (5.0)	14 (4.3)	
Faculty member housing	2 (0.6)	0 (0.0)	2 (0.6)	
**Marital status**				
Married	9 (2.5)	0 (0.0)	9 (2.8)	
Single	345 (97)	21 (100)	315 (97)	1.00
Divorced	1 (0.3)	0 (0.0)	1 (0.3)	

Data are presented as means ± SDs; other non-normal variables are presented as medians (Quartiles 1–3). *P* values were obtained using the independent sample t-test and the Mann-Whitney U test for normal and non-normal variables, respectively. N (%) was used for categorical variables. *P* values for categorical variables were obtained using Fisher's exact test. $P < 0.05$ was considered significant.

Table S2. Indicators of vitamin B12 serum by quartiles among women of childbearing age

	**Quartile 1 (≤305.8 pmol/l)**		**Quartile 2 (305.9 - 398.9 pmol/l)**		**Quartile 3 (399.0 - 534.6 pmol/l)**	
	OR (95%CI)	P-value	OR (95%CI)	P-value	OR (95%CI)	P-value
Age (y)	1.04 (0.84 - 1.30)	0.71	1.00 (0.79 - 1.26)	0.98	0.92 (0.71 - 1.19)	0.52
BMI (kg/m2)	1.02 (0.96 - 1.09)	0.53	1.01 (0.94 - 1.08)	0.77	1.00 (0.93 - 1.08)	0.99
Glucose level (> 5.6 mmol/L)	0.61 (0.18 - 2.03)	0.42	0.96 (0.30 - 3.06)	0.94	2.19 (0.77 - 6.21)	0.14
VB12 intake (> 2.4 mcg/d)	0.28 (0.05 - 1.75)	0.17	0.22 (0.04 - 1.32)	0.09	1.81 (0.16 - 20.15)	0.62
Using protein supplement	0.32 (0.02 - 4.30)	0.39	0.40 (0.03 - 5.33)	0.48	3.56 (0.61 - 20.68)	0.15
Protein intake (> 46 g/d)	0.40 (0.03 - 4.59)	0.46	0.91 (0.07 - 12.00)	0.94	0.36 (0.02 - 5.73)	0.47
Coffee intake(> 750 mL/d)	2.00 (0.32 - 12.40)	0.45	1.73 (0.26 - 11.43)	0.57	0.47 (0.04 - 5.61)	0.55
Income (< 10,000 SAR)	2.01 (0.77 - 5.23)	0.15	2.06 (0.78 - 5.45)	0.14	1.84 (0.68 - 4.98)	0.22

Sitting time (≥ 7 h/d)	1.89 (0.94 - 3.78)	0.07	0.70 (0.34 - 1.44)	0.33	1.25 (0.61 - 2.59)	0.54
Vigorous physical activity (≥ 60 min/w)	0.95 (0.39 - 2.33)	0.90	1.35 (0.55 - 3.35)	0.51	0.67 (0.24 - 1.88)	0.44

OR's are obtained from multinomial regression analysis while taking Quartile 4 (>534.6 pmol/l) as reference category
